# Diterpenoids from the Buds of *Pinus banksiana* Lamb

**DOI:** 10.3390/molecules17089716

**Published:** 2012-08-13

**Authors:** Patricia Georges, Jean Legault, Serge Lavoie, Carole Grenon, André Pichette

**Affiliations:** Laboratoire d'analyse et de séparation des essences végétales, Département des sciences fondamentales, Chaire de recherche sur les agents anticancéreux d'origine naturelle, Université du Québec à Chicoutimi, Québec, G7H 2B1 Canada; Email: pgeorges@univ-ag.fr (P.G.); jean_legault@uqac.ca (J.L.); serge_lavoie@uqac.ca (S.L.); carole_grenon@uqac.ca (C.G.)

**Keywords:** *Pinus banksiana*, bud, dehydroabietane, cytotoxicity, antibacterial activity

## Abstract

Three new diterpenoids, namely 7α-hydroxyabieta-8,11,13,15-tetraen-18-oic acid, 7β,15,18-trihydroxyabieta-8,11,13-triene, 13,15-dihydroxypodocarpa-8,11,13-triene, and 12 other known compounds were isolated from buds of *Pinus banksiana* Lamb. All these compounds, except for 7-oxodehydroabietinol, were isolated for the first time from this plant. Their structures were elucidated by detailed spectroscopic studies and comparison with published data. All isolated compounds were tested for cytotoxic and antibacterial activities. Overall, two compounds, 7-oxodehydroabietinol and 18-nor-4,15-dihydroxyabieta-8,11,13-trien-7-one, showed moderate cytotoxicity against a human lung carcinoma cell line.

## 1. Introduction

*Pinus banksiana* Lamb (jack pine) is widely distributed in North American forests and particularly in Canada, where its presence extends from Cape Breton Island, Nova Scotia, up to the Mackenzie river in the Northwest Territories [1,2,3]. Although jack pine was essentially used in the wood industry as a source of pulpwood, lumber, and round timber [3], it was also used in traditional medicines. Gum, when chewed, can fight colds [4], inner bark, soaked and softened, has been used as a poultice to heal wounds [5] and leaves were used as a fuminant to revive comatose patient and to clear congested lungs [6]. The pine oil and pine tar have been also used to make disinfectants, antiseptics and insecticides [7]. Numerous studies realized on bark, wood, needles, resin and essential oils of *Pinus banksiana* have reported the presence of monoterpenoids [8,9], diterpenoids [8,9,10,11], sesquiterpenoids [8,12], triterpenoids [11], phenylpropanes [13], flavonoids, lignans and stilbenes [10,14]. However, to this day, no study of the cytotoxic and antibacterial activities of the buds of jack pine was made.

**Figure 1:**
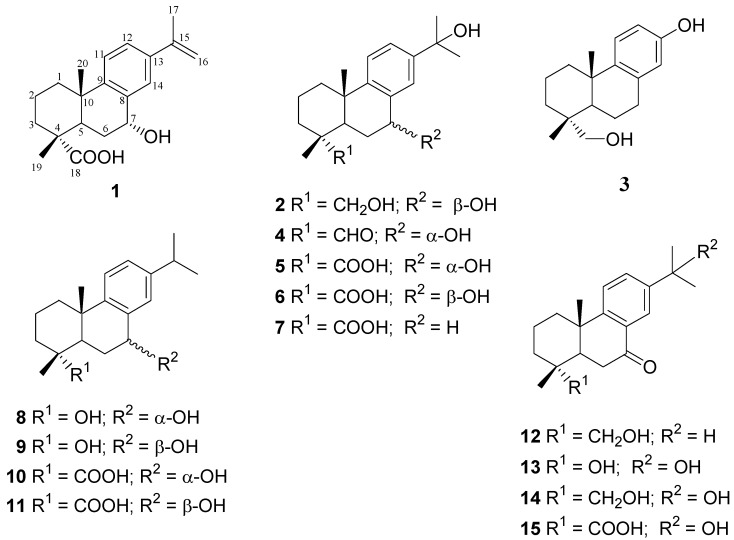
Diterpenes from buds of *Pinus banksiana*.

Phytochemical investigation of the buds of jack pine resulted in the isolation of 15 compounds (see Figure 1), including three new diterpenoid derivatives: 7*α*-hydroxyabieta-8,11,13,15-tetraen-18-oic acid (**1**), 7*β*,15,18-trihydroxyabieta-8,11,13-triene (**2**) and 13,15-dihydroxypodocarpa-8,11,13-triene (**3**). The known compounds were identified as 7*α*,15-dihydroxyabieta-8,11,13-trien-18-al (**4**) [15], 7*α*,15-dihydroxydehydroabietic acid (**5**) [16], 7*β*,15-dihydroxydehydroabietic acid (**6**) [17,18], 15-hydroxydehydroabietic acid (**7**) [19], 18-nor-abieta-8,11,13-triene-4,7α-diol (**8**) [20], 18-nor-abieta-8,11,13-triene-4,7*β*-diol (**9**) [21], 7*α*-hydroxydehydroabietic acid (**10**) [17], 7*β*-hydroxydehydroabietic acid (**11**) [17,22], 7-oxodehydroabietinol (**12**) [23], 18-nor-4,15-dihydroxyabieta-8,11,13-trien-7-one (**13**) [15,23], 15,18-dihydroxyabieta-8,11,13-trien-7-one (**14**) [24] and 15-hydroxy-7-oxo-abieta-8,11,13-trien-18-oic acid (**15**) [25]. Compound **9** is a known synthetic dehydroabietane derivative that is now identified for the first time as a natural product. The structure elucidations of **1**–**3** were based on spectroscopic analyses, including 1D and 2D NMR spectroscopic techniques.

## 2. Results and Discussion

*P. banksiana* buds were extracted successively with hexanes, CH_2_Cl_2_ and MeOH. After solvent evaporation, each extract was investigated for *in vitro* cytotoxic and antibacterial activities. Cytotoxic activity evaluations were carried out on lung cancer (A549), colorectal cancer (DLD-1) and normal skin fibroblasts (WS1) human cell lines using the Hoechst assay [26]. Antibacterial activity was evaluated against *Escherichia coli* and *Staphylococcus aureus*. Results displayed in Table 1 show that the hexanes extract exerted a moderate activity against A549 (IC_50_, 45 ± 4 µg/mL), DLD-1 (IC_50_, 44 ± 3 µg/mL) cell lines and its antibacterial activity against *S. aureus* was interesting (IC_50_, 29 ± 3 μg/mL). The CH_2_Cl_2_ extract showed a significant cytotoxic activity against A549 (IC_50_, 26 ± 3 µg/mL), DLD-1 (IC_50_, 32 ± 3 µg/mL) cell lines and a weak activity on *S. aureus* (64 ± 5 µg/mL). Finally, the MeOH extract exhibited no activity. Therefore, following studies were focused on the CH_2_Cl_2_ extract that was further separated over column chromatography on silica gel to afford eleven fractions (A–K). All fractions activities were tested against both cancer cell lines and bacterial strains. Only fraction F, G and H were found strongly cytotoxic activity against A549 with IC_50_ values ranging from 5 to 7 µg/mL and against DLD-1 with IC_50_ values ranging from 12 to 14 µg/mL. Interestingly, these fractions were found selective against cancer cell lines in comparison with normal cells (IC_50_, 39 to 79 µg/mL). Remaining fractions were found inactive with IC_50_ values greater than 100 µM. Fraction F was active against *S. aureus* (IC_50_, 51 ± 2 µg/mL) while fractions G, H and K were found inactive (IC_50_ > 100 µM). In a next step, fractions F, G, H and K were separated by a combination of chromatographic procedures to afford the three new compounds **1**–**3** together with 12 known compounds. The structures of the new compounds were determined as follows and the known products were identified by comparison of their spectroscopic data with values found in the literature.

**Table 1:** Cytotoxic and antibacterial activities of extracts and fractions.

Compounds	IC_50_ (µg/mL) ^a,b^				
	A549 ^c^	DLD-1 ^d^	WS1 ^e^	*S. aureus * ^f^	*E. coli* ^g^
Hexanes extract	45 ± 4	44 ± 3	59 ± 6	29 ± 3	>100
CH_2_Cl_2_ extract	26 ± 3	32 ± 3	42 ± 4	64 ± 5	>100
MeOH extract	>100	>100	>100	>100	>100
Fraction F	7 ± 2	12 ± 2	79 ± 3	51 ± 2	>100
Fraction G	5 ± 1	14 ± 1	42 ± 3	>100	>100
Fraction H	6 ± 1	13 ± 1	39 ± 3	>100	>100
Fraction K	84 ± 1	>100	>100	>100	>100
Etoposide ^h^	0.4 ± 0.2	1.7 ± 0.3	5.9 ± 0.6		
Chloramphenicol ^i^				>1.6	0.12 ± 0.02

^a^ Mean values for three independent assays; ^b^ Concentration inhibiting 50% cell growth; ^c^ A549, human lung carcinoma cell line; ^d^ DLD-1, human colorectal adenocarcinoma cell line; ^e^ WS1, human normal skin fibroblasts; ^f^ *Staphylococcus aureus* ATCC 25923; ^g^ *Escherichia coli* ATCC 25922; ^h^ Positive control for cytotoxicity assay; ^i^ Positive control for antibacterial assay.

**Figure 2:**
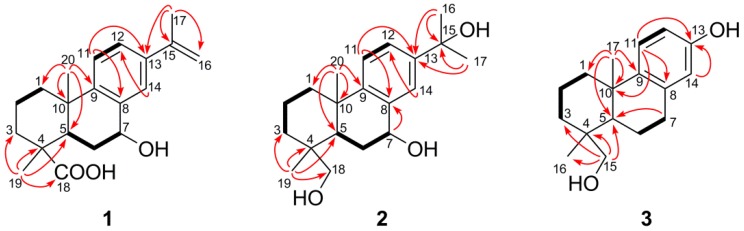
Key ^1^H–^1^H COSY (▬) and HMBC (H
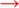
C) correlations of compounds **1**–**3**.

Compound **1**, a white amorphous powder, was assigned as C_20_H_26_O_3_ by positive HRESIMS (*m*/*z* 337.1766 calcd for C_20_H_26_O_3_Na^+^ 337.1774). Its IR spectrum showed the presence of a hydroxyl (3391 cm^−1^) and an olefinic bond (1639 cm^−1^). The ^1^H-NMR spectrum (Table 2) showed an AMX aromatic ring system [δ_H_ 7.46 (1H, d, *J* = 1.6 Hz, H-14), 7.39 (1H, dd, *J* = 8.4, 1.6 Hz, H-12) and 7.23 (1H, d, *J* = 8.4 Hz, H-11)], an oxymethine proton at δ_H_ 4.81 (1H, d, *J* = 3.5 Hz, H-7), an isopropenyl group [δ_H_ 2.14 (3H, s, Me-17), 5.06 (1H, s, H-16Z) and 5.36 (1H, s, H-16E)] and two methyl group singlets [δ_H_ 1.18 (3H, s, Me-20) and 1.30 (3H, s, Me-19)]. The ^13^C-NMR spectrum of **1** (Table 3) showed a signal characteristic of a carboxylic acid group (δ_C_ 182.3, C-18), eight unsaturated carbons (δ_C_ 148.3, 142.6, 139.1, 135.8, 127.5, 125.6, 124.2, 112.1) and one hydroxylated carbon (δ_C_ 68.2, C-7). HMBC correlations (see Figure 2) observed between H_3_-19 and C-3, C-4, C-5 and C-18 (δ_C_ 36.3, 46.9, 39.6, 182.3, respectively), together with the presence of an aromatic ring, suggested that the molecule was a dehydroabietane [17]. The hydroxyl group was assigned at position 7 because of its ^1^H-^1^H COSY correlation with H-6 (δ_H_ 1.72 and 2.14, both overlapped) (see Figure 2). 

**Table 2:** ^1^H-NMR data for compounds **1**, **2** and **3 **(400 MHz, ^a^ in CDCl_3_, ^b^ in CD_3_OD, *J* in Hz).

Position	1 ^a^	2 ^b^	3 ^a^
1	2.33 (*m*)	2.27 (*br d* , 12.6)	2.26 (*m*)
	1.51 (*m*)	1.35	1.30 (*m*)
2	1.79 (*m*)	1.81	1.79 (*m*)
		1.69	1.64 (*m*)
3	1.80 (*m*)	1.48	1.51 (*m*)
		1.36	1.31 (*m*)
5	2.51 (*m*)	1.76	1.64 (*m*)
6	2.14 (*m*)	2.20 (*dd*, 11.0, 6.7)	1.80 (*m*)
	1.72 (*m*)		1.65
7	4.81 (*d*, 3.5)	4.86 (*t*, 8.9)	2.79
11	7.23 (*d*, 8.4)	7.21 (*d*, 8.3)	7.04 (*d*, 8.6)
12	7.39 (*dd*, 8.4, 1.6)	7.31 (*dd*, 8.3, 0.9)	6.51 (*dd*, 8.6, 2.7)
14	7.46 (*d*, 1.6)	7.64 (*d*, 1.9)	6.42 (*d*, 2.7)
15			3.43 (*d* , 7.5)
			3.09 (*d* , 7.5)
16	5.36 (*s*)	1.57 (*s*)	0.84 (*s*)
	5.06 (*s*)		
17	2.14 (*s*)	1.57 (*s*)	1.17 (*s*)
18		3.50 (*d* , 10.9)	
		3.20 (*d* , 10.9)	
19	1.30 (*s*)	0.89 (*s*)	
20	1.18 (*s*)	1.29 (*s*)	

**Table 3:** ^13^C-NMR data for compounds **1**, **2** and **3** (100 MHz, ^a^ in CDCl_3_, ^b^ in CD_3_OD).

Position	1 ^a^	2 ^b^	3 ^a^
1	37.6 (*t*)	38.3 (*t*)	40.0 (*t*)
2	18.5 (*t*)	18.5 (*t*)	19.9 (*t*)
3	36.3 (*t*)	34.7 (*t*)	36.3 (*t*)
4	46.9 (*s*)	37.5 (*s*)	38.9 (*s*)
5	39.6 (*d*)	42.3 (*d*)	45.1 (*d*)
6	31.4 (*t*)	29.8 (*t*)	19.9 (*t*)
7	68.2 (*d*)	70.9 (*d*)	31.2 (*t*)
8	135.8 (*s*)	137.6 (*s*)	137.5 (*s*)
9	148.3(*s*)	148.1 (*s*)	142.7 (*s*)
10	37.6 (s)	38.1 (*s*)	38.2 (*s*)
11	124.2 (*d*)	124.4 (*d*)	126.4 (*d*)
12	125.6 (*d*)	123.9 (*d*)	113.9 (*d*)
13	139.1 (*s*)	146.5 (*s*)	155.5 (*s*)
14	127.5 (*d*)	123.1 (*d*)	115.7 (*d*)
15	142.6 (*s*)	72.5 (*s*)	72.0 (*t*)
16	112.1 (*t*)	31.7 (*q*)	18.0 (*q*)
17	21.8 (*q*)	31.6 (*q*)	26.0 (*q*)
18	182.3 (*s*)	71.6 (*t*)	
19	16.3 (*q*)	17.5 (*q*)	
20	24.1 (*q*)	25.7 (*q*)	

The multiplicity of H-7 was a broad doublet (*J* = 3.5 Hz) suggesting that the configuration is α. The configurations of other stereocenters were assessed by comparing the ^13^C chemical shifts of **1** with those of 7α-hydroxydehydroabietic acid [17]. Hence, compound **1** was established as 7*α*-hydroxyabieta-8,11,13,15-tetraen-18-oic acid.

**Figure 3:**
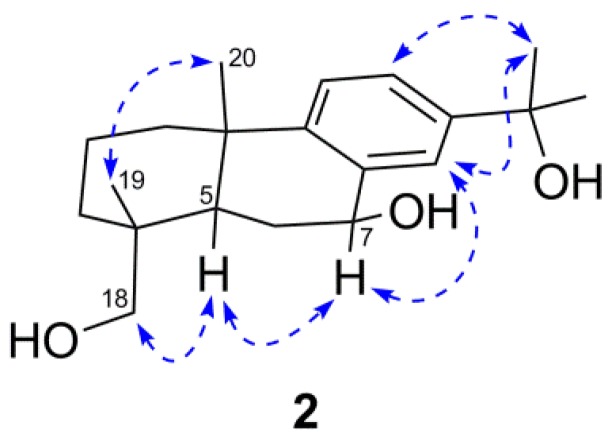
Key NOESY (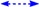) correlations of compound **2**.

Compound **2** was assigned the molecular formula C_20_H_30_O_3_, as established from its HRESIMS (*m*/*z* 341.2077 calcd for C_20_H_30_O_3_Na^+^ 341.2087). The IR spectrum showed absorbances consistent with hydroxyl (3411 cm^−1^) and olefinic (1650 cm^−1^) groups. The ^1^H-NMR spectrum of **2** (Table 2) showed the presence of three AMX aromatic protons [δ_H_ 7.64 (1H, d, *J* = 1.9 Hz, H-14), 7.31 (1H, dd, *J* = 8.3, 1.9 Hz, H-12), 7.21 (1H, d, *J* = 8.3 Hz, H-11)], one oxygenated methine at δ_H_ 4.86 (1H, t, *J* = 8.9 Hz, H-7), one oxygenated methylene [δ_H_ 3.50, 3.20 (each 1H, d, *J* = 10.9 Hz, H-18a,b)], and three singlets methyls [δ_H_ 1.57 (6H, s, Me-16 and 17), 1.29, 0.89 (each 3H, s, Me-20 and 18)]. ^13^C-NMR signals (Table 3) were observed at δ_C_ 72.5 (C-15), 70.9 (C-7) and 71.6 (C-18) for tertiary, secondary and primary alcohol groups respectively, along with six other signals assigned to an aromatic ring. The methyl groups Me-16 and Me-17 showed HMBC correlations with C-15 and C-13 (δ_C_ 146.5), indicative of a hydroxypropyl branched at C-13 (see Figure 2). Long-range correlations were observed between H_3_-19 and H_2_-18 with C-3, C-4 and C-5 (34.7, 37.5, 42.3, respectively) suggesting that the hydroxyl function of the primary alcohol was attached to C-18. The β-configuration of the hydroxyl group at C-7 was determined from the ^1^H-NMR spectrum, in which a triplet at δ_H_ 4.86 (*J* = 8.9 Hz) due to an axial proton was observed [18]. Cross peaks in the NOESY spectrum (see Figure 3) were observed between H_3_-19 and H_3_-20 and between H_2_-18 and H-5, supporting the relative stereochemistry depicted. Thus, compound **2** was identifed as 7*β*,15,18-trihydroxyabieta-8,11,13-triene.

Compound **3** has the molecular formula, C_17_H_24_O_2_, as shown from its positive HRESIMS at *m*/*z* 283.1670 (calcd for C_17_H_24_O_2_Na^+^ 283.1668). Analysis of the ^1^H-NMR spectrum (Table 2) indicated the presence of three protons in a trisubstituted aromatic ring [δ_H_ 7.04 (1H, d, *J* = 8.6 Hz), 6.51 (1H, dd, *J* = 8.6, 2.7 Hz) and 6.42 (1H, d, *J* = 2.7 Hz)], one oxygenated methylene [δ_H_ 3.43 and 3.09 (2H, d, *J* = 7.5 Hz)], and two singlets methyls at δ_H_ 1.17 and 0.84 (both 3H, s, Me-17 and Me-16, respectively). The ^13^C-NMR spectrum (Table 3) showed 17 carbon signals, including three olefinic carbons and two oxygenated carbons. The resonances at δ_H_ 3.43, 3.09 and δ_C_ 72.0 support the presence of a secondary alcohol group. Long-range correlations from H_2_-15 with C-3, C-4, C-5 and C-16 where observed in the HMBC spectrum (see Figure 2), indicating the position of the alcohol group. The ^13^C signal at δ_C_ 155.5 suggested the presence of a phenolic group. The location of this hydroxyl group at C-13 was confirmed by observation of C–H long range correlations from H-11 to C-8, C-10 and C-13 in the HMBC spectrum. The presence of the two alcohols was confirmed by the absorption bands at 3448 cm^−1^ in the FTIR spectrum of **3**. These data, together with other results of COSY and HMBC analysis (see Figure 2), confirmed that compound **3** is 13,15-dihydroxypodocarpa-8,11,13-triene.

Cytotoxic and antibacterial activities of all compounds were evaluated against two human cancer cell lines, lung carcinoma (A549) and colorectal adenocarcinoma (DLD-1) and two bacterial strains, *S. aureus* and *E. coli* (Table 4). With the exception of compounds **12** and **13**, all compounds were found inactive against both cancer cell lines tested, IC_50_ > 100 µM. Compound **12** exhibited a moderate cytotoxicity against A549 and DLD-1 with IC_50_ of 34 and 47 µM, respectively. Compound **13** was also found active against A549 (IC_50_, 46 µM) but not to DLD-1. These results are in good agreement with those reported by Barrero [27] for compound **12**. As far as antibacterial activity was concerned, all compounds were inactive which is also in agreement with previously published results for compounds **7**, **11**, **12** and **15** [28,29,30]. Compounds **12** and **13** were in part responsible of the cytotoxic activity of the CH_2_Cl_2_ extract of bud from *P. banksiana*. However, additional studies will be conducted to identify other active compounds.

**Table 4:** *In vitro *cytotoxicity and antibacterial activities from isolated compounds isolated from fractions F, G and H.

Compounds	IC_50_ (µM) ^a,b^				
	A549 ^c^	DLD-1 ^d^	WS1 ^e^	*S. aureus * ^f^	*E. coli * ^g^
**1**	>100	>100	>100	>100	>100
**2**	>100	>100	>100	>100	>100
**3**	>100	>100	>100	>100	>100
**4**	>100	>100	>100	>100	>100
**5**	>100	>100	>100	>100	>100
**6**	>100	>100	>100	>100	>100
**7**	98 ± 2	>100	>100	>100	>100
**8**	63 ± 7	91 ± 9	>100	>100	>100
**9**	>100	>100	>100	>100	>100
**10**	>100	>100	>100	>100	>100
**11**	>100	>100	>100	>100	>100
**12**	34 ± 4	47 ± 9	>100	>100	>100
**13**	46 ± 3	>100	>100	>100	>100
**14**	>100	>100	>100	>100	>100
**15**	>100	>100	>100	>100	>100
Etoposide ^h^	0.7 ± 0.3	2.9 ± 0.5	10 ±2		
Chloramphenicol^i^				>5	0.37 ± 0.06

^a ^Mean values for three independent assays; ^b^ Concentration inhibiting 50% cell growth; ^c^ A549, human lung carcinoma cell line; ^d^ DLD-1, human colorectal adenocarcinoma cell line; ^e^ WS1, human normal skin fibroblasts; ^f^ *Staphylococcus aureus* ATCC 25923; ^g^ *Escherichia coli* ATCC 25922; ^h^ Positive control for cytotoxicity assay; ^i^ Positive control for antibacterial assay.

## 3. Experimental

### 3.1. General

Optical rotations were measured with an automatic polarimeter Rudolph Research Analytical Autopol IV. High resolution electrospray ionization mass spectrum was conducted in positive mode with an Applied Biosystems/MDS Sciex QSTARXL QqTOF MS system. FTIR spectra were recorded with a Perkin–Elmer SpectrumOne. The 1D and 2D NMR spectra (^1^H–^1^H COSY, HSQC and HMBC) were performed using an Avance 400 Bruker spectrometer equipped with a 5 mm QNP-probe. Chemical shifts were expressed in δ (ppm) units relative to TMS as an internal standard and coupling constants were given in Hertz. The analytical HPLC separations were performed using an Agilent 1100 series instrument fitted with a UV-Vis diode array detector and a MS detector Agilent G1946 VL together with an atmospheric pressure chemical ionization (APCI) source. Preparative HPLC was performed on an Agilent 1100 liquid chromatography system, equipped with a solvent delivery system, an autosampler and UV-MWD detector. The column configuration consisted of an Intertsil prep-ODS C18 column (6.0 × 250 mm; 10 µm) for analytical analysis and an Intertsil prep-ODS C18 column (20 × 250 mm; 10 µm) for preparative HPLC. Column chromatographic separations were carried out using silica gel (40–63 µm with indicator F_254_, Silicycle, Québec, Canada) and C_18_ reversed phase silica gel (carbon 11%, 40–69 μm, Silicycle). High performance flash chromatography was performed using a HPFC-Analogix F12-40 system equipped with a silica gel column C18, 40 µM (silicycle, Québec, Canada). Analytical thin-layer chromatography was performed with silica gel 60 F_254_, 0.25 mm pre-coated TLC plates (Silicycle). Diterpene compounds were detected by spraying TLC plates with vanillin-sulfuric acid reagent followed by heating at 110 °C. The yields were calculated from the weight of dry plant material.

### 3.2. Plant Material

Buds of *Pinus banksiana* were collected in the boreal forest of the Saguenay region (Quebec, Canada) in May 2007. The specimen was identified by Patrick Nadeau (Université du Québec à Chicoutimi) and a voucher specimen (QFA-0540468) was deposited at the Herbarium Louis-Marie of Université Laval, Québec, Canada.

### 3.3. Extraction and Isolation

Air-dried buds of *Pinus banksiana* (1.6 kg) were reduced to powder before being successively extracted with hexanes, CH_2_Cl_2 _and MeOH using a Soxhlet apparatus (5 L each for 48 h). Evaporation under reduced pressure, at a temperature not exceeding 45 °C, yielded hexanes (489 g), CH_2_Cl_2_ (84 g) and MeOH (166 g) extracts. The CH_2_Cl_2_ extract was subjected to silica gel column chromatography using a gradient of CHCl_3_-MeOH (60:1, 25:1 and 0:100) to give 11 fractions A–K. Fraction F (7 g) was purified on silica gel CC, eluted with CH_2_Cl_2_–EtOAc gradient (20:1→5:1) and 11 fractions were obtained. Subfr. F9 (690 mg) was submitted to reversed-phase flash chromatography using a gradient MeOH–H_2_O (70:30→80:20) as eluent, to give 10 fractions. Subfr. F9.5 was separated by preparative HPLC using an isocratic mobile phase of MeOH–H_2_O (75:30, during 40 min) to afford **12** (6 mg). Fraction G (8 g) was separated on silica gel column, eluted with CHCl_3_–EtOAc gradient (5:1→5:5) to give **7** (880 mg) and eight fractions. Subfr. G5 was purified on reversed-phase CC with MeOH-H_2_O (75:30, 80:20 and 100:0) as eluent to afford **3** (6 mg). Fraction H (7 g) was chromatographed on silica gel CC with a gradient elution of CH_2_Cl_2_–EtOAc (2.5:1→0.5:1) to give 14 fractions. Fr. H10 (280 mg) and H11 (260 mg) were combined and separated on reversed-phase CC eluted with MeOH–H_2_O (50:50→75:25) to yield 11 subfractions. Subfr. H10.4 was purified by preparative HPLC using an isocratic mobile phase of MeOH–H_2_O (55:45, during 40 min) to afford **13** (8 mg). Preparative HPLC purification performed on fractions H10.6 and H10.7 using an isocratic mobile phase consisting of MeOH–H_2_O (60:40, during 50 min) gave **14** (19 mg) and **4** (6 mg). Subfr. H10.9 was purified by preparative HPLC with a gradient elution of MeOH-H_2_O (65:35, during 50 min) to give **9** (5 mg). Fractions H12 (460 mg) and H13 (570 mg) were combined and submitted to flash chromatography on reversed-phase eluted with a gradient elution of MeOH–H_2_O (60:40→78:22). Eleven fractions were obtained. Preparative HPLC purification performed on fraction H12.7 using an isocratic mobile phase consisting of MeCN–H_2_O (45:55, during 60 min) afforded **8** (10 mg) and **11** (19 mg). Fraction H14 (3 g) was submitted to high performance flash chromatography using MeOH–H_2_O gradient (68:32→74:26) as eluent. Eleven fractions were obtained. Subfr. H14.2 was purified by preparative HPLC with an isocratic mobile phase of MeOH–H_2_O–HCOOH (60:40:0.1, during 40 min) to yield **15** (54.8 mg). Preparative HPLC purification performed on Subfr. H14.8 using an isocratic mobile phase of MeOH–H_2_O–HCOOH (75:25:0.1, during 52 min) gave **1** (10 mg) and **10** (29 mg). Fraction K (6 g) was separated on silica gel column eluted with CH_2_Cl_2_–MeOH gradient (15:1→5:1) to give 5 fractions. 3 g of subfr. K4 was chromatographed on silica gel CC, eluted with EtOAc–MeOH (90:0.1 and 90:1) to give **2** (10 mg). A preparative HPLC of 700 mg of subfr. K4, with MeCN–H_2_O–HCOOH (30:70:0.1→42:58:0.1, during 25 min and 30:70:0.1, during 8 min) as eluent, permitted to obtained **5** (20 mg) and **6** (44 mg).

### 3.4. Compound Characterization

*7α-Hydroxyabieta-8,11,13,15-tetraen-18-oic acid* (**1**). White amorphous powder; [α]_D_^25^ +28.5° (*c* 0.70, MeOH); IR (neat) *v*_max_ 3391, 2930, 1639, 667 cm^−1^; HR-ESIMS *m*/*z* 337.1767 [M+Na]^+^ (calcd for C_20_H_26_O_3_Na, 337.1774); for ^1^H, ^13^C-NMR spectroscopic data, see Table 2 and Table 3.

*7β,15,18-Trihydroxyabieta-8,11,13-triene* (**2**). White amorphous powder; [α]_D_^25^ −15.3° (*c* 0.50, MeOH); IR (neat) *v*_max_ 3411, 2951, 2929, 2850, 2119, 1650, 1452, 1380, 1015 cm^−1^; HRESIMS *m*/*z* 341.2077 [M+Na]^+^ (calcd for C_20_H_30_O_3_Na, 341.2087); for ^1^H, ^13^C NMR spectroscopic data, see Table 2 and Table 3.

*13,15-Dihydroxypodocarpa-8,11,13-triene * (**3**). White amorphous powder; [α]_D_^25^ +12.9° (*c* 0.24, MeOH); IR (neat) *v*_max_ 3430, 2931, 2096, 1641, 1497, 1451, 1381, 1242 cm^−1^; HRESIMS *m*/*z* 283.1670 [M+Na]^+^ (calcd for C_17_H_24_O_2_Na, 283.1668); for ^1^H, ^13^C NMR spectroscopic data, see Table 2 and Table 3.

### 3.5. Cell Lines and Culture Conditions

Lung carcinoma (A549), colorectal adenocarcinoma (DLD-1) and normal skin fibroblast (WS1) human cell lines were obtained from the American Type Culture Collection (ATCC). All cell lines were cultured in minimum essential medium containing Earle’s salts and L-glutamine (Mediatech Cellgro, VA, USA), to which were added 10% fetal bovine serum (Hyclone), vitamins (1X), penicillin (100 I.U./mL) and streptomycin (100 µg/mL), essential amino acids (1X) and sodium pyruvate (1X) (Mediatech Cellgro, VA). Cells were kept at 37 °C in a humidified environment containing 5% CO_2_. Antibacterial activity was tested on *Escherichia coli *ATCC 25922 and *Staphylococcus aureus *ATCC 25923E.

### 3.6. Cytotoxicity Assay

Exponentially growing cells were plated on 96-well microplates (BD Falcon) at a density of 5 × 10^3^ cells per well in 100 μL of culture medium (DMEM with 10% SVF) and were allowed to adhere for 24 h before treatment. Increasing concentrations of each compound in MeOH or DMSO were then added (100 μL per well) and cells were incubated for 48 h. The final concentration of MeOH or DMSO in the culture medium was maintained at 0.25% (v/v) to avoid solvent toxicity. Microplates were then emptied and stored at −80 °C for 24 h. In a next step, 100 μL of SDS (0.01%) were added and the microplates were incubated at room temperature during 3 h before being put back to the cold. After 24 h, cells were prepared for cellular DNA assay with 100 μL of Hoechst dye 33342. Measurements were performed on the same labsystems at 365 and 460 nm wavelengths. Survival percentage was defined as the fluorescene in experimental wells compared to the control wells after subtraction of the blank values. Etoposide was used as positive control. Each experiment was carried out two times in triplicata. IC_50_ results were expressed as averaged values and the corresponding standard deviations were computed.

### 3.7. Antibacterial Assay

Antibacterial activity was evaluated using the microdilution method described by Banfi *et al*. [31] with some modifications. Exponentially growing bacteria were plated in 96-well flat bottom microplates (BD Flacon) at a density of 5 × 10^3^ gram-negative *E. coli *(ATCC 25922) or 40 × 10^3^ gram-positive *S. aureus *(ATCC 25923) per well in 100 μL nutrient broth (Difco). The concentration of ethanol in the culture medium was maintained at 0.25% (v/v) to avoid solvent toxicity. Thereafter, 50 μL of 4% resazurin was added to each well and the microplates were incubated for 6 h at 37 °C. Fluorescence was measured after 6 h with an automated 96-well Fluoroskan Ascent Fl™ plate reader (Labsystems) using 530 and 590 nm excitation and emission wavelengths.

## 4. Conclusions

Three new diterpenoids, namely 7*α*-hydroxyabieta-8,11,13,15-tetraen-18-oic acid (**1**), 7*β*,15,18-trihydroxyabieta-8,11,13-triene (**2**), 13,15-dihydroxypodocarpa-8,11,13-triene (**3**), and twelve known compounds, were isolated from the buds of *Pinus banksiana* Lamb. All isolated compounds were tested for their antibacterial and cytotoxic activities, and among all the isolated compounds, only compounds, 7-oxo dehydroabietinol (**12**) and 18-nor-4,15-dihydroxyabieta-8,11,13-trien-7-one (**13**), showed moderate cytotoxicity against lung carcinoma A549. From the above results, the cytotoxic activities of the isolated compounds do not explain the strong activities of the CH_2_Cl_2_ extract and further studies are needed to identify the active cytotoxic compounds from CH2Cl2 extract or other extracts.
